# Inhibition of Retinoblastoma mRNA Degradation through Poly (A) Involved in the Neuroprotective Effect of Berberine against Cerebral Ischemia

**DOI:** 10.1371/journal.pone.0090850

**Published:** 2014-03-06

**Authors:** Yu-Shuang Chai, Zhi-Yi Yuan, Fan Lei, Yu-Gang Wang, Jun Hu, Feng Du, Xi Lu, Jing-Fei Jiang, Dong-Ming Xing, Li-Jun Du

**Affiliations:** 1 MOE Key Laboratory of Protein Sciences, Laboratory of Molecular Pharmacology and Pharmaceutical Sciences, School of Life Sciences and School of Medicine, Tsinghua University, Beijing, China; 2 Department of Neuro-Oncology, MD Anderson Cancer Center, University of Texas, Houston, Texas, United States of America; 3 Department of Mathematics, Tulane University, New Orleans, Louisiana, United States of America; Institute of Molecular and Cell Biology, Singapore

## Abstract

Berberine is one kind of isoquinoline alkaloid with anti-apoptotic effects on the neurons suffering ischemia. To address the explanation for these activities, the berberine-induced cell cycle arrest during neurons suffering ischemia/reperfusion had been studied in the present study. According to the *in vitro* neurons with oxygen-glucose deprivation and *in vivo* ICR mice with cerebral ischemia/reperfusion, it was found that berberine could protect the mRNA of retinoblastoma (Rb) from degradation through its function on the poly(A) tail. The prolonged half-life of retinoblastoma 1 (gene of Rb, RB1) mRNA level secures the protein level of retinoblastoma, which facilitates cell cycle arrest of neurons in the process of ischemia/reperfusion and subsequently avoids cells entering in the apoptotic process. The poly(A) tail of RB1 mRNA, as a newly identified target of berberine, could help people focus on the interaction between berberine and mRNA to further understand the biological activities and functions of berberine.

## Introduction

Ischemic stroke is one of the leading causes of death in many countries [1]. It causes a series of complex responses, including neurotransmitter release, oxidative stress, inflammatory response, reactive oxygen species production [2], specific receptor activation, gene expression change, and neuron death [3], [4]. Protection of neurons from cell death, especially the neurons of the hippocampus that is highly sensitive to ischemic injury, is the major therapeutic strategy for stroke [5]–[7]. Neuron is a kind of terminally differentiated cells which is closely related to the arrest of the cell cycle [8] and the reentry of the neuron cell cycle could result in apoptosis [9]. For the reported correlation between cell cycle reentry and neuron apoptosis after ischemia/reperfusion injury [10], [11], inhibition of cell cycle reentry might be one important strategy assisting neurons survival in cerebral ischemia.

Retinoblastoma (Rb) is reported to be important in the maintenance of cells in the terminal differentiated state by arresting cells at G1 phase [12]–[15]. Acute Rb family inactivation could force neurons underwent S-phase progression, indicating the potential involvement of Rb in the cell cycle reentry [16]. Berberine (BBR) possesses many pharmacological activities [17]. In the process of ischemia/reperfusion, BBR could down-regulate the caspase 3 and NF-κB to suppress the pro-apoptosis signal. Meanwhile, BBR can also stimulate the expression of PI3K p55γ and promote the phosphorylation of BCL-2, AKT, GSK, and CREB, which are essential for cell survival [18], [19]. However, BBR can also act as an antitumor compound inducing cell apoptosis through the cell cycle inhibition [20]–[23]. Considering that the cell cycle reentry is the hallmark of neurons entering apoptosis in the process of ischemia/reperfusion, the checkpoint of cell cycle might be the effective target of BBR on neural cells.

The present work identified that retinoblastoma protein is the target of BBR during cerebral ischemia/reperfusion. BBR acts on the poly (A) tail of RB1 (gene of Rb) mRNA to antagonize its degradation. The stabilized the level of Rb protein effectively prevented the cell cycle reentry of neuron and assisted neurons survival during cerebral ischemia injury.

## Materials and Methods

### Animals

The male ICR mice (8–10 week old, weighing 21–23 g) and the pregnant SD rats used in this study were purchased from Vital River Laboratories (Beijing, China) and kept in the animal center of Tsinghua University. Mice were maintained under standard temperature and pressure with 12 h light/dark cycle at a controlled temperature (25°C) and relative humidity (45–55%) with access to standard food pellets and tap water ad libitum. All studies were conducted under protocols approved by the Institutional Animal Care and Use Committee of Tsinghua University and the Animal Welfare and Ethics Committee of Tsinghua University (Approval ID: 2013-DuLJ001).

### Dosages and Groups

In the *in vivo* study, the mice were randomly divided into 5 groups of six mice in each group. One group served as the normal control and was subjected to a sham operation. The remaining four groups served as the models, one group served as the model control and the remaining three groups were given BBR via three dosages, 1, 2, and 4 mg/kg, by intraperitoneal injection (i. p.). Normal saline injections were used as controls in both normal and model groups.

### Experimental Procedures

#### Cytotoxicity assay

The cytotoxicity of BBR in PC12 cells was measured using an MTT (3-(4,5-dimethylthiazol-2-yl)- 2,5-diphenyltetrazolium bromide) assay, as described previously [18].

*Analysis of cell cycle and apoptosis by flow cytometry* A Calibur Flow Cytometer system (BD Inc., U.S.) was used to analyze the cell cycle distribution and apoptosis. The PC12 cells used for detection of cell cycle distribution were stained with propidium iodide (PI). A FITC Annexin V Apoptosis Detection Kit I (BD Pharmingen, U.S.) was used to detect apoptosis.

#### Separation of primary neurons

The separation of primary neurons of neonatal rats was performed as described previously [18]. The separated primary neurons were adjusted to about 1×10^6^ cells/ml with 1 ml inoculated into each well of a 6-well plate precoated with poly-D-lysine (50 µg/ml; Sigma, U.S.). The culture medium was changed every 48 h. The purity of neurons was over 95% after 8 days, as examined by Map2 antibody (Abcam, U.K.). The primary neurons were used after 8 days of culturing [24].

#### Oxygen-glucose deprivation in vitro

Oxygen-glucose deprivation (OGD) followed by reperfusion may mimic the pathological conditions of ischemia *in vitro*. In the OGD model, the culture medium was replaced with glucose-free Earle’s solution, as described previously [24]. It was done in a humidified atmosphere of 95% N_2_ and 5% CO_2_ at 37°C for 2 h. Then the normal medium was replaced for another 4 h under normal conditions as reperfusion.

#### Model of cerebral ischemia-reperfusion in vivo

The cerebral ischemia and reperfusion of mice was performed by ligaturing the bilateral carotid arteries as described previously [18]. Phenobarbital (50 mg/kg, i. p.) served as anesthetic for mice. During the operation, both common carotid arteries were ligatured for 5 min, and then allowed to relax for 10 min. This process was repeated twice. The arteries were relaxed after the last ligature for reperfusion.

### Western Blot Analysis

Protein concentrations were determined using a Protein Assay Kit (Zhongsheng Biotech., China). First, 100 µg total protein was loaded onto SDS-PAGE gels (10%) using a previously described protocol [18]. The intensity of targeted proteins was measured using Quantity One software (Bio-Rad). β-actin served as an internal control. The primary antibodies for phosphorylation of retinoblastoma protein (phosphor-Rb, p-Rb) S795, S780 and S807/811 were purchased from Cell Signaling Technology (U.S.) [25], [26]. Primary antibody for Rb was purchased from Abcam (U.S.). Primary antibody for β-actin and the second antibody with horseradish peroxidase (HRP)-conjugated were purchased from ZSGB-Bio (Beijing, China).

### Construction of RB1 Knockdown Stable Cell Lines using Lentiviral Vectors

The lentiviral vectors PLL3.7, pMD2.G, pMDLg-pRRE, and pRSV-Rev were provided by Dr. Ya-Dong Hu, School of Life Sciences, Tsinghua University. A multiple cloning site was engineered and placed immediately after the U6 promoter. HpaI and XhoI were used as digestion sites. Oligo sequences were as follows: sense: 5′-TGGAGCACGAGTGTAATGTATTCAAGAGATACATTACACTCGTGCTCCTTTTTC-3′; antisense: 5′-TCGAGAAAAAGGAGCACGAGTGTAATGTATCTCTTGAATACATTACACTCGTGCTCCA-3′. A lentiviral stock (containing the packaged transfer vector) needed to be produced by co-transfecting the optimized packaging plasmid mix (pMD2.G, pMDLg-pRRE, and pRSV-Rev) and the transfer vector (PLL3.7) containing the shRNA of RB1 into the HEK293T cell line to produce a replication-incompetent lentivirus [27]. PC12 cells were transfected with the lentivirus. The stable PC12 cell lines with RB1 knockdown were produced by sorting with a BD FACSAriaII according to the expression of GFP.

### Real Time PCR

The total RNA from PC12 was extracted using the RNA pre-pure Cell/Bacteria Kit (Tiangen Biotech, China). The concentrations of the total RNA were measured using a Gene Quant 100 Spectrophotometer (General Electric Company, Germany). Five micrograms of total RNA were used to generate complementary DNA using an anchored oligo (dT)_18_ primer and EasyScript Reverse Transcriptase enzyme (Transgene, China). The cDNA was subsequently used as a template for real-time PCR using SYBR Green Master Mix (Tiangen Biotech, China) according to the manufacturer’s instructions. The primer sequences of RB1 were as follows (NCBI Reference Sequence: NM_009029.2): forward: 5′-TGCTGAAGGCGGCAATCCCC-3′; reverse: 5′- CGAGTCAGGTGTCCCGAGGGT-3′.

### Constructing RB1 Promoter-GFP Plasmid

The RB1 promoter gene was obtained using PCR from the genome, which was extracted from the PC12 cells using TIANamp Genomic DNA Kit (Tiangen Biotech., China). pEGFP-N1 plasmid was provided by Professor Ye-Guang Chen, School of Life Sciences, Tsinghua University. The CMV promoter in pEGFP-N1 plasmid was replaced by the RB1 promoter using the restriction enzymes AseI and PstI. The extent of RB1 promoter was determined by the expression of GFP measuring by a BD Calibur Flow Cytometer system (BD Inc, U.S.). The primer sequences of RB1 promoter were as follows: forward: 5′-GAGCATGTCTAGTTATTAATATCTTTGTAGCTGGACCTGGGCCT-3′; reverse: 5′-GTGACGTCACTGCAGGGGAGCCAGCGAGCTGTGGAG-3′.

### Electrophoretic Mobility Shift Assay (EMSA)

Nuclear extracts of PC12 cells were prepared using the NE-PER Nuclear and Cytoplastic Extraction Reagent Kit according to the manufacturer’s instructions (Pierce, U.S.). The GC box-containing oligonucleotide was synthesized as complementary oligo-deoxyribo-nucleotide strands and labeled using a Biotin 3′ End Labeling Kit (Thermo, U.S.). The GC box probe sequence was: 5′-CTCGCCCCGCCCCGATCGAAT-3 ′. The combination between the GC box binding protein (SP1, specificity protein-1) and GC box was assessed using a Light Shift Chemiluminescent EMSA Kit (Pierce, U.S.). The labeled GC box probes were incubated with different concentrations of BBR before the binding reaction. The binding reaction and gel detection were performed according to the manufacturer’s instructions.

### Chromatin Immunoprecipitation (ChIP)

Chromatin was prepared using the EZ-ChIP Chromatin Immunoprecipitation Kit (Millipore, U.S.) according to the manufacturer’s guidelines. The content of GC box-containing sequences in the RB1 promoter that were associated with SP1 protein was analyzed using agarose gel electrophoresis and real-time PCR using SYBR Green Master Mix (Tiangen Biotech, China) according to the manufacturer’s instructions. The primer sequences used with the RB1 promoter were as follows: forward: 5′-AATGGCCCCACCCTGGACCG-3′; reverse: 5′-GCGGCCTCTGGGAGAACGCT-3′. The antibody for SP1 used in ChIP was purchased from Santa Cruz (U.S.).

### Construction of the RB1-promoter-RB1 mRNA-GFP and RB1-promoter-RB1 mRNA-GFP-Δpoly (A) Plasmids

The plasmids RB1-promoter-GFP and RB1-promoter-GFP-*Δ*polyA were provided by Dr. Zhi-Yi Yuan, School of Life Sciences, Tsinghua University. BamHI and KpnI were used as digestion sites. The primers for RB1 with the BamHI and KpnI restriction sites were as follows: forward: GGGGCTCGAGGGTACCATGCCGCCCAAAACCCCCCGAAAAACGGCC; reverse: GTGACGTCAGGATCCCGTTTCTCTTCCTTGTTTGAGGTATCCATGCT. The cells transfected with plasmids were sorted using a BD FACS Aria II (BD Inc., U.S.).

### Fluorospectrophotometry for Poly (A)

The poly (A) has a length of 30 nt, it was synthesized by Sigma-Aldrich (U.S.). The concentration of berberine was 0.5 µg/ml and the concentration of the poly (A) was 40 µM. The volume of the reaction system was 20 µl. The 384-Well black microplate was purchased from Corning (U.S., product #3540). The fluorescence spectra (Excitation wavelength: 350 nm; emission wavelengths: 400 nm–700 nm [28]) were detected by Multifunctional Microplate Reader (Thermo Scientific, U.S.).

### Data Analysis

All data were expressed as mean ± S.D. Data were statistically analyzed using one-way analysis of variance (ANOVA). The F test was carried out using Excel Software for Office 2007 (Microsoft, U.S.). After the F test, the student’s *t*-t test between two groups was carried out. *P*<0.05 was considered statistically significant.

## Results

### Anti-apoptotic Effect of BBR by Rb Activation Resulting in Cell Cycle Arrest in vitro

**Figure 1:**
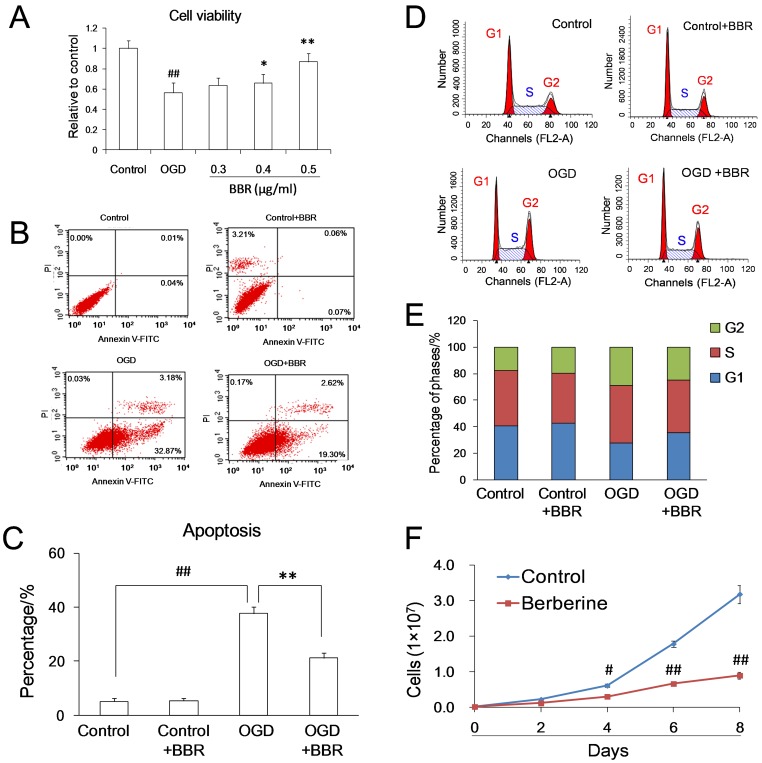
Protective effects of berberine (BBR) in PC12 cells subjected to oxygen and glucose deprivation (OGD). (A) Protective effect of different concentrations of BBR in PC12 cells after OGD as indicated by MTT assay. (B)–(C) Anti-apoptotic effects of BBR in PC12 cells subjected to OGD as indicated using Annexin V-FITC double staining and flow cytometry. (D)–(E) Effects of BBR on cell cycle arrest in PC12 cells subjected to OGD as indicated by flow cytometry. (F) The cell growth curves with and without BBR in different days. The final concentration of BBR was 0.5 µg/ml. BBR was added to the culture 1 h before OGD treatment and throughout reperfusion. # P<0.05 v.s. the control, ## P<0.01 v.s. the control; *P<0.05, **<0.01 v.s. the OGD. Data are presented as mean ± S.D. of six independent experiments (n = 6).

With the dosage of 0.5 µg/ml, BBR was found to protect PC12 cells from OGD damage (Fig. 1A) and to reduce the percentage of apoptotic cells caused by OGD damage (Figs. 1B–C), which was in accordance with previous report [29]. After OGD damage, the percentage of cells in G1 phase of control groups dropped from 40.69% to 27.77%, and the percentage of cells in G1 phase of BBR groups kept at 35.63% (Figs. 1 D–E). BBR could slow down the cellular growth (Fig. 1F). All these suggested that the effects of BBR arresting cells at G1 phase and lowering cell’s proliferation potentially contribute to its protective effects on neurons suffering OGD damage.

**Figure 2:**
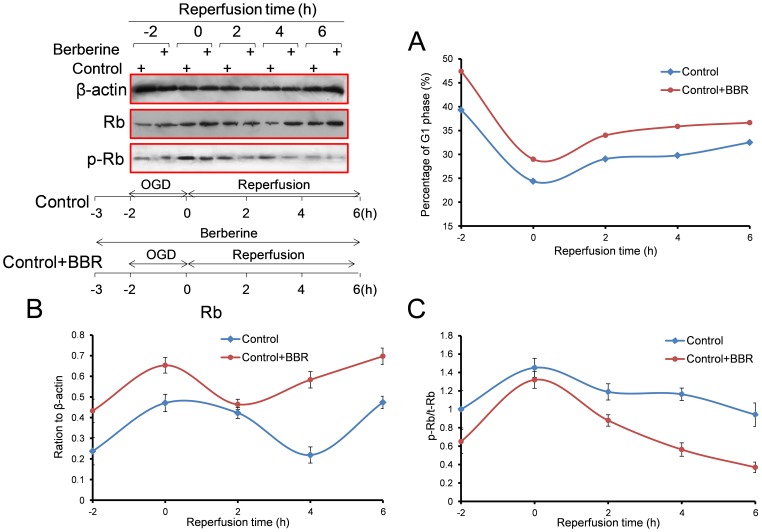
Effect of berberine (BBR) on the cell cycles and Rb expressions in PC12 cells during the 6 h of reperfusion after 2 h of OGD. (A) Expression of Rb proteins and time schedule of the experiment. (B) The percentage of cells in the G1 phase. (C) Total Rb protein. (D) Ratio of phosphor-Rb (S795) to total Rb (phosphor-Rb/Rb). Berberine (0.5 µg/ml) was added to the culture 1 h before OGD treatment and throughout reperfusion. Data are presented as mean ± S.D. of three independent experiments (n = 3).

In the process of OGD in PC12 cells, the percentage of G1-phase cells decreased and total Rb protein increased. BBR was found to enhance Rb and increase the percentage of G1-phase cells (Fig. 2A). As the reperfusion continued, it was found that BBR could increase the percentage of G1-phase cells (Fig. 2B) and the expression of Rb (Fig. 2C), as well as reduce the phosphor-Rb (S795) and the ratio of phosphor-Rb/Rb (Fig. 2D).

**Figure 3:**
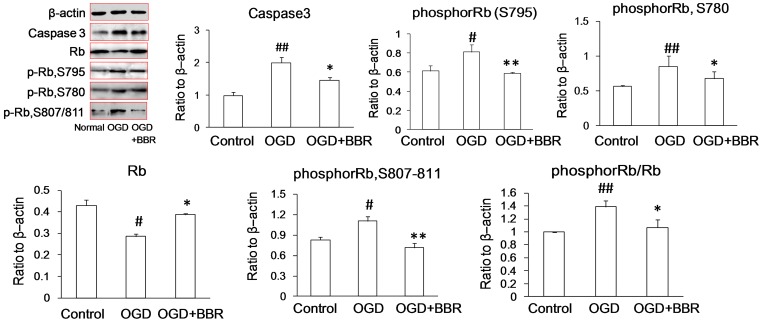
Expressions of Rb and phosphor-Rb (p-Rb) in primary neurons as measured by Western blot. Berberine (BBR) (0.5 µg/ml) was added in the culture 1 h before OGD treatment and throughout reperfusion. Data are presented as mean ± S.D. of three independent experiments (n = 3). # P<0.05, ## P<0.01 v.s. normal groups; *P<0.05, **P<0.01 v.s. OGD groups.

During OGD damage in primary neurons, the expression of Rb protein was down-regulated and phosphor-Rb (S795, S780, S807/811) and phosphor-Rb/Rb were up-regulated significantly. However, BBR could increase the expression of Rb, decreased the level of expression of phosphor-Rb, and thereby decrease the ratio of phosphor-Rb to Rb (Fig. 3), which is in accordance with the results observed in PC12 cells.

### Anti-apoptotic Effects of BBR by Rb Activation in vivo

**Figure 4:**
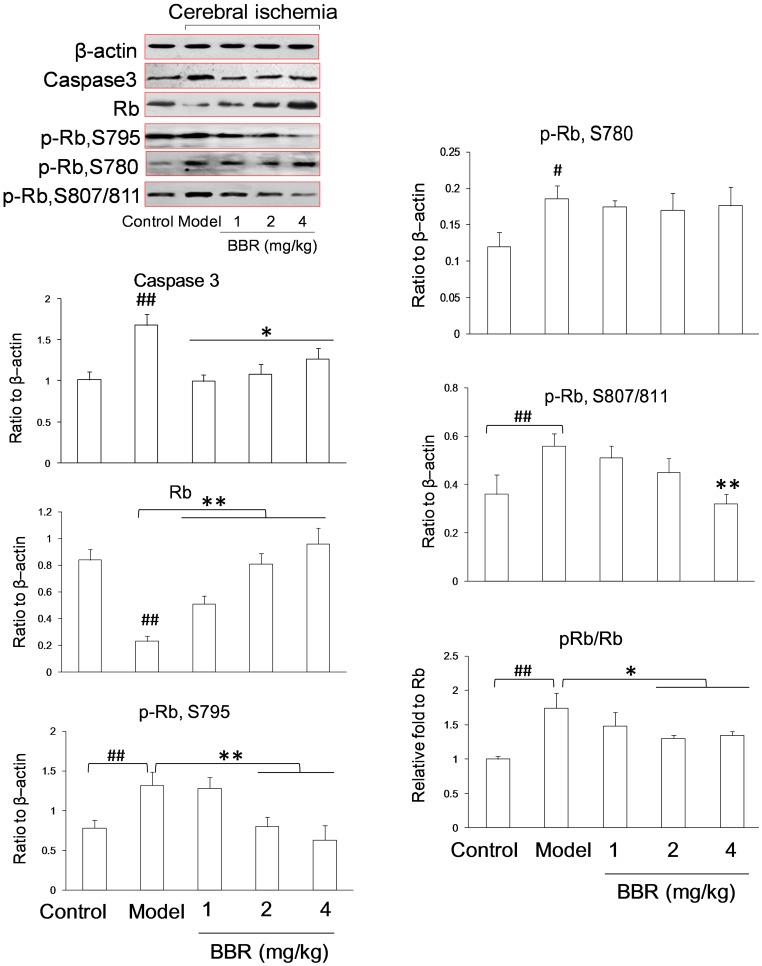
Expressions of Rb and phosphor-Rb (p-Rb) in the hippocampus of cerebral ischemia-treated mice as measured by Western blot. Berberine (BBR) was administrated by intraperitoneal injection with three different dosages (1, 2 and 4 mg/kg). Data are presented as mean ± S.D. of six mice (n = 6). ## P<0.01 v.s. control groups; *P<0.05, **P<0.01 v.s. model groups.

BBR was found to antagonize the degradation of Rb and the phosphorylation of Rb protein in OGD process, which could subsequently affect the cell cycle, rendering the cells resistant to OGD injury *in vitro*. To further confirm the effect of BBR on Rb, an *in vivo* ischemia/reperfusion mice model was studied. Western blot analysis showed total Rb protein to be up-regulated and phosphor-Rb (S795, S780, S807/811) and phosphor-Rb/Rb to be down-regulated in the presence of BBR in a dose-dependent manner (Fig. 4), which were consistent with those of the *in vitro* experiments.

### Protective Effect of BBR by Targeting RB1

**Figure 5:**
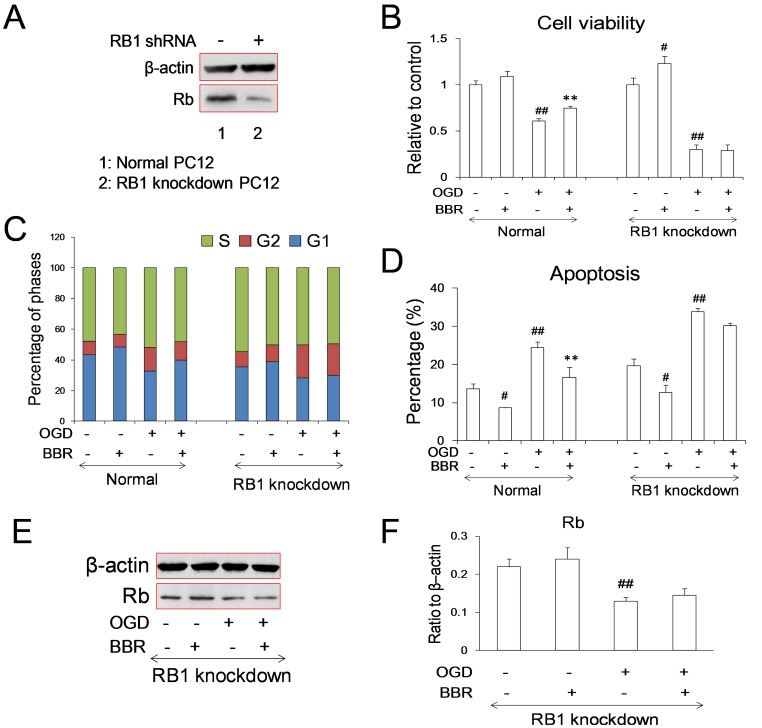
Effects of berberine (BBR) on the PC12 cells with RB1 knockdown. (A) Expression of Rb in normal and RB1 knockdown cells. RB1shRNA was constructed to lentiviral vector PLL3.7. (B) Cell viabilities of normal and RB1 knockdown cells. Columns represent the mean ± S.D. ## P<0.01 v.s. control groups; *P<0.05 v.s. model groups (n = 6). (C) Phases of cell cycle were detected in normal and RB1 knockdown cells. (D) Apoptosis of BBR after OGD treated cells using Annexin V-FITC double staining were detected by using flow cytometry. Data are presented as mean ± S.D. ## P<0.01 v.s. control groups; *P<0.05, **P<0.01 v.s. the OGD (n = 6). (E)–(F) Expression of Rb in cells infected with carrying RB1 shRNAlentivirus under normal and OGD conditions. BBR (0.5 µg/ml) was added in the culture 1 h before OGD treatment and throughout reperfusion. Data are presented as mean ± S.D. of three independent experiments (n = 3). ## P<0.01 v.s. the normal; **P<0.01 v.s. the OGD.

In order to confirm the importance of Rb in cerebral ischemic injury and determine the protective effects of BBR during stroke, RB1 knockdown stable cell lines were constructed using lentiviral vectors. In the presence of OGD damage, the protective effect of BBR on OGD suffering PC12 cells was abolished with the RB1 knockdown (Figs. 5A–B). The percentage of G1 phase cells was also lower in RB1 knockdown cells, which was in accordance with previous reports [30], [31]. And the effect of BBR on PC12 cells arresting at G1 phase could only observed in cells without RB1 knockdown (Fig. 5C), confirming the key role of Rb in the cell cycle reentry and the cell cycle arresting effect of BBR during OGD process.

Consistent with the correlation between cell cycle reentry and neuronal apoptosis, more apoptosis were observed in RB1 knockdown cells after OGD treatment. The anti-apoptosis effect of BBR on cells was also abolished by the RB1 knockdown (Fig. 5D). And meanwhile, the regulation of Rb expression by BBR was disappeared in RB1 knockdown cells during both normal and OGD conditions (Figs. 5E–F). All these further supported the conclusion that RB1 is the target of BBR in the process of OGD.

### Effect of BBR on the Activity of RB1 Promoter

**Figure 6:**
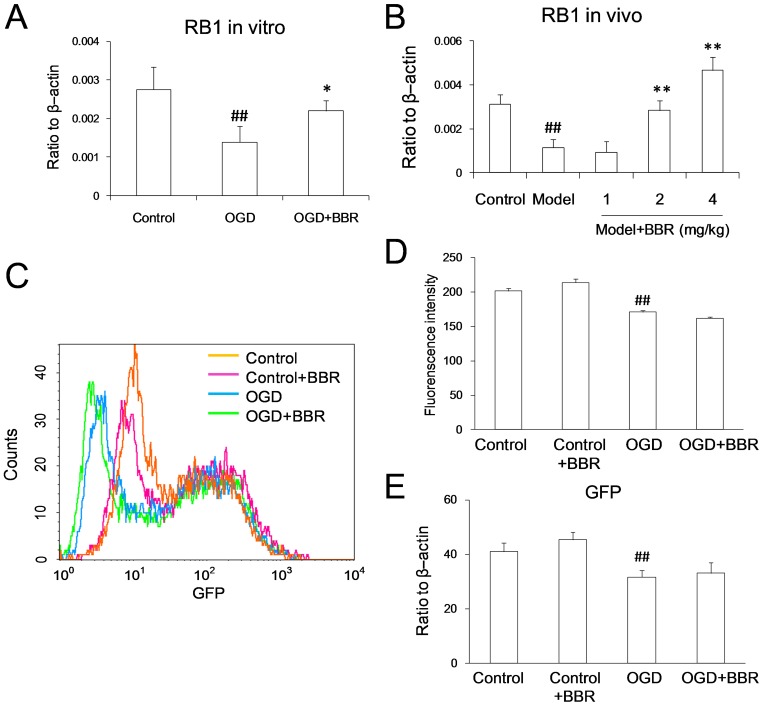
Berberine (BBR) up-regulated the RB1 mRNA, but couldn’t influence the activity of RB1 promoter. (A) RB1 mRNA level in primary neurons as measured using real-time PCR. BBR (0.5 µg/ml) was added in the culture 1 h before OGD treatment and throughout the reperfusion time. Data represent the mean ± S.D. of three independent experiments (n = 3). # P<0.05, ## P<0.01 v.s. the normal; *P<0.05, **P<0.01 v.s. the OGD. (B) RB1 mRNA level in the hippocampus of cerebral ischemia mice as measured using real-time PCR. BBR was administrated by intraperitoneal injection with three different dosages (1, 2, 4 mg/kg). Data represent the mean ± S.D. of six mice (n = 6). # P<0.05, ## P<0.01 v.s. the control; *P<0.05, **P<0.01 v.s. the model. (C)–(E) RB1 promoter. (C) Overlay of the fluorescence intensity of GFP with RB1 promoter. (D) Quantity of the fluorescence intensity of GFP. (E) GFP mRNA level started by RB1 promoter detected using real-time PCR. Data are presented as mean ± S.D. of three independent experiments (n = 3). ## P<0.01 v.s. the control.

Gene transcription is the first step of protein expression. Because of the described antagonist effect of BBR on the degradation of Rb during OGD conditions, real-time PCR was used to verify the potentially regulatory effect of BBR on the transcription of RB1. With the *in vitro* OGD model, BBR was noted that to be able to antagonize the down-regulation of RB1 mRNA level (Fig. 6A). And the *in vivo* studies further confirmed the antagonism effect of BBR on RB1 mRNA down-regulation (Fig. 6B).

To clarify the antagonist effect of BBR on RB1 mRNA down-regulation was due to the increasing mRNA expression or inhibition of mRNA degradation, RB-promoter-GFP plasmid was constructed to determine the effect of BBR on the transcriptional activity of RB1 promoter. In accordance with the Rb protein level, the intensity of GFP fluorescence was reduced significantly when cell suffering OGD injury, but BBR had no effect on this reduction (Figs. 6C–D). The expression of GFP mRNA was reduced also during OGD conditions, BBR had no effect on this injury, which coordinated with the result of GFP fluorescence previously (Fig. 6E).

**Figure 7:**
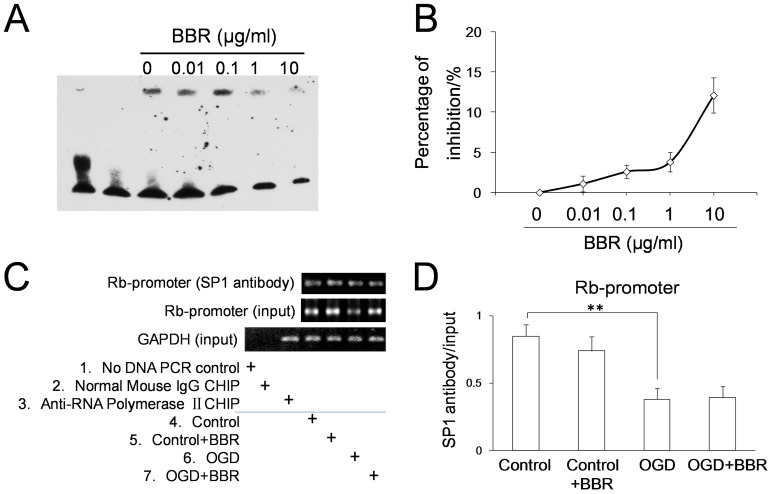
Effects of berberine (BBR) on the combination of SP1 protein with the GC box in RB1 promoter. (A) Image was taken by using EMSA. (B) Suppressive curve of BBR on the combination between SP1 protein and GC box. (C) PCR detection of the GC box-containing DNA fragment of the RB1 promoter bound by SP1 protein in living cells using ChIP. (D) The quantitative analysis of the GC box-containing DNA fragment of the RB1 promoter combined by SP1 detected using real-time PCR. BBR was 0.5 µg/ml. Data are presented as mean ± S.D. of three independent experiments (n = 3). **P<0.01.

In order to determine whether BBR acted on the DNA sequence of transcription resulting in RB1 transcription, the experiments of EMSA in genomic sequence and ChIP in living cells were conducted. The EMSA results showed that concentrations of BBR below 1 µg/ml did not interfere with the ability of specificity protein-1 (SP1) associating with the GC box on RB1 promoter *in vitro* (Figs. 7A–B). And ChIP assay results showed that, either in normal conditions or in OGD conditions, there was no effect of BBR on the interaction of SP1 and GC box on RB1 promoter (Figs. 7C–D). All these data suggested that BBR has no effect on the transcription of RB1 promoter.

### Effect of BBR on RB1 mRNA Degradation

**Figure 8:**
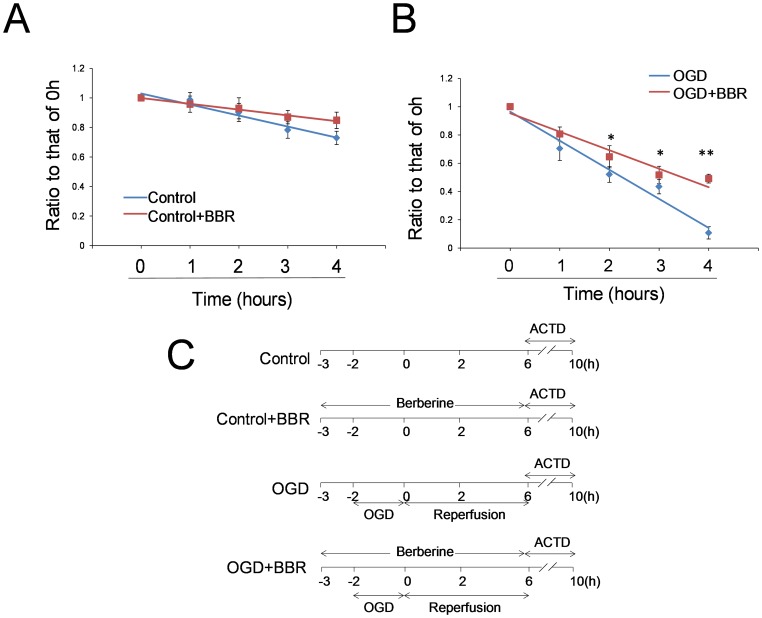
Effects of berberine (BBR) on the degradation of RB1 mRNA in normal and OGD conditions. (A) The time-dependent degradation of RB1 mRNA level detected using real-time PCR. (B) The time-dependent degradation of RB1 mRNA level with OGD detected using real-time PCR. (C) The experimental schedule. BBR (0.5 µg/ml) was added in the culture 1 h before OGD treatment and throughout the reperfusion time. Actinomycin D (ACTD) was 2 µg/ml. After ACTD added in culture, the cells were harvested at 0 h, 1 h, 2 h, 3 h, and 4 h for RNA detection. Data were presented as ratio to that of 0 h (ΔCp/Cp0 h). The linear equations are as follows: (A) Control, y = −0.075x+1.104, R2 = 0.9505; Control+BBR, y = −0.039x+1.038, R2 = 0.9839. (B) OGD, y = −0.206x+1.17, R2 = 0.9679; OGD+BBR, y = −0.131x+1.085, R2 = 0.9439. Data are presented as mean ± S.D. from three independent experiments (n = 3). *P<0.05, **P<0.01 v.s. OGD groups.

Since BBR has no effect on the transcription of RB1 mRNA, the suppression mRNA degradation is the other reasonable explanation for the higher RB1 mRNA in the cells protected by BBR during OGD conditions. Real-time PCR assay was used to determine whether BBR could affect the degradation of RB1 mRNA. Gene transcription inhibition by Actinomycin D assay was employed for the study of RNA degradation. The results revealed that BBR could attenuate the degradation of RB1 mRNA under normal (Fig. 8A) and OGD conditions (Fig. 8B). Under the OGD conditioning, the degradation of RB1 mRNA was more obvious than that in normal conditions. BBR could distinctly inhibit the degradation of RB1 mRNA in OGD conditions. All these suggested that the effect of BBR on RB1 was achieved by protecting RB1 mRNA from degradation.

### Suppression of BBR against RB1 mRNA Degradation Through its Function on the Poly (A)

**Figure 9:**
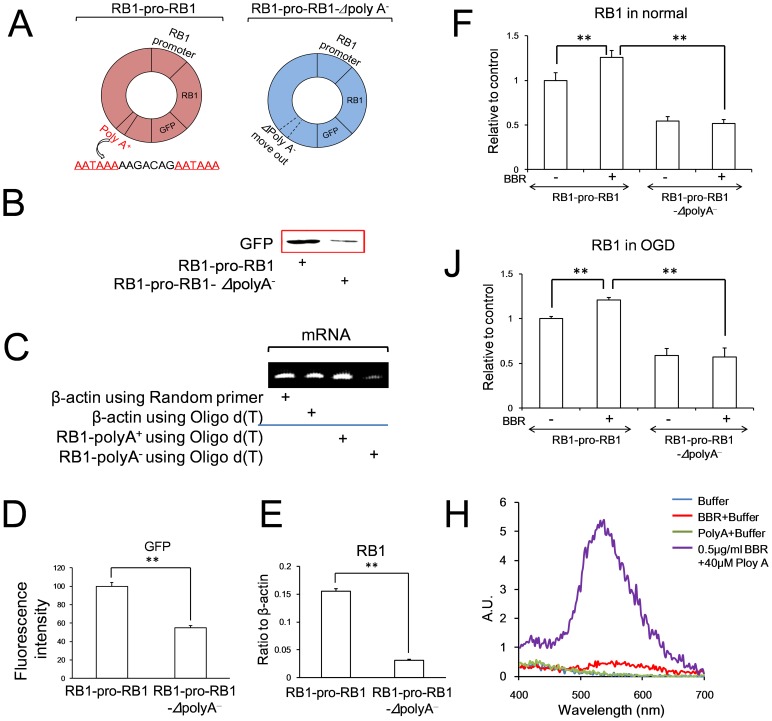
Effects of berberine (BBR) to inhibit the degradation of RB1 mRNA with poly(A) (RB1-pro-RB1) or without poly(A) (RB1-pro-RB1-Δpoly A-). (A) Cartoon of the plasmid models used in this study. Polyadenylation signals were showed in red. (B) Expressions of GFP in cells with transfection of RB1-promoter-RB1-GFP with and without poly(A) plasmids detected using Western blot. (C) Expressions of β-actin and RB1 mRNA using random primer and oligo d(T) in the cells with transfection of RB1-promoter-RB1-GFP with and without poly(A) plasmids detected using RT PCR. (D) Fluorescence intensity in cells with transfection of RB1-promoter-RB1-GFP with and without poly(A) plasmids detected using flow cytometry. (E) RB1 mRNA expressions using oligo d(T) as the primer in cells with transfection of RB1-promoter-RB1-GFP with and without poly(A) plasmids detected using real time PCR. (F) RB1 mRNA level in PC12 cells in normal conditions with transfection of RB1-promoter-RB1-GFP with and without poly(A) plasmids administrated with or without BBR. (J) RB1 mRNA level in PC12 cells in oxygen and glucose deprivation (OGD) conditions with transfection of RB1-promoter-RB1-GFP with and without poly(A) plasmids administrated with or without BBR. (H) The fluorescence spectrum of BBR associating with poly(A) (40 µM). BBR was 0.5 µg/ml. Data are presented as mean ± S.D. from three independent experiments (n = 3). **P<0.01.

Because of the reported thymine and adenine preference when BBR binding to nucleotides [32], the poly (A) tail of RB1 mRNA, which is important to the mRNA stability, was studied. Cell lines harboring RB1-promoter-RB1 mRNA-GFP-poly (A) and RB1-promoter-RB1 mRNA-GFP-*Δ*poly (A)^−^ (polyadenylation was removed) were constructed (Fig. 9A). Because of the instability of RB1 mRNA, GFP expression was much weaker in cells with the RB1-promoter-RB1 mRNA-GFP-*Δ*poly (A) ^−^ than in cells with RB1-promoter-RB1 mRNA-GFP-poly (A) (Figs. 9B and 9D). For Oligo d(T) combining with poly(A) specificity [33], the expression of RB1 mRNA in cells with the RB1-promoter-RB1 mRNA-GFP-*Δ*poly(A) ^−^ was lower obviously than that in cells with RB1-promoter-RB1 mRNA-GFP-poly(A) (Figs. 9C and 9E), conforming the poly (A) tail of RB1 had been removed in RB1-promoter-RB1 mRNA-GFP-*Δ*poly(A)^−^. In PC12 cells, BBR could enhance the expression of RB1 mRNA with poly (A) tail either in normal conditions or in OGD conditions, but couldn’t inhibit the degradation of the expression of RB1 mRNA without poly(A) tail in both normal and OGD conditions (Figs. 9F and 9J), which suggested that poly(A) is the target of BBR for the stability of RB1 mRNA. To confirm the effect of BBR, the artificial polyadenylation [poly (A)] was synthesized to study the directly the physical interaction between poly (A) tail and BBR *in vitro*. The increasing fluorescent intensity of BBR confirmed the direct interaction between BBR and the poly(A), suggesting that the poly(A) tail located the 3′UTR of RB1 mRNA is the target for BBR protection of RB1 mRNA from degradation in OGD process (Fig. 9H).

## Discussion

Cell cycle reentry is a hot issue in the field of cerebral ischemia/reperfusion research [34]. When neurons suffering ischemia/reperfusion injury, the terminal differentiated cells would go to the S phase or G2/M phase from G1 phase but cannot finish the cell division, which would be followed by cellular apoptosis [35], [36]. Preventing cell cycle reentry after neurons suffering ischemia damage is viewed as a rational therapeutic strategy for stroke therapy. The present study showed that the neuronal protective effect of BBR in the process of ischemia/reperfusion through keeping neuron arrest at G0/G1 phase correlating with up-expression of Rb protein in *in vitro* and *in vivo* experiments. BBR was found to act directly on the poly (A) tail, preventing mRNA degradation and facilitating stable Rb expression.

Retinoblastoma (Rb) is a tumor suppressor gene playing important role in cell cycle regulation in proliferation, apoptosis, and neuron survival [37]–[41]. In proliferating cells, cyclins/CDKs could phosphorylate Rb protein, which in turn acts as a transcriptional factor and regulates its downstream gene expression for cell cycle process [42]. In the present work, BBR was found to increase the expression of Rb protein and decrease the expression of phosphor-Rb, which is opposite to the proliferative signal and consequently arrests the cell cycle, confirming the protective role of BBR on neurons during ischemia/reperfusion is correlated with the retinoblastoma-dependent cell cycle regulation. Our previous study showed that BBR could affect the cell cycle of neurons in the process of cerebral ischemic injury [43]. BBR could reduce the protein and mRNA levels of cyclinD1 and CDK4. Therefore, BBR inhibited the forming of cyclinD1/CDK4 complex, which in turn attenuated the phosphorylation of Rb protein.

The transcription of RB1 gene is mainly regulated by transcriptional factor of specificity protein 1 (SP1) [44], [45]. Due to the present results, BBR was unable to neither activate the transcription of the RB1 promoter nor influence the association of SP1 with RB1 promoter, as measured in living cells. The results of EMSA *in vitro* showed that BBR, with the dosage of 10 µg/ml, could inhibit the binding of SP1 protein to the GC box (percentage of the inhibition was about 13%). Previous results have shown that BBR could disturb cell viability at more than 1 µg/ml [18]. Therefore, the limitation of toxic free intracellular concentrations of BBR in living cells restricts its ability of suppressing the association of SP1 with the GC box on RB1 promoter.

The results of present study revealed the inhibitory effect of BBR on RB1 mRNA degradation in OGD conditions. Previous study reported that the poly(A) tail located at the 3′UTR plays an important role in maintaining the stability of mRNA [46], and BBR could bind to RNA directly *in vitro* with adenine and uracil preference [27], [47]. Based on the *in vitro* fluorescent screening, the directly physical interaction between BBR and poly (A) tail on RB1 mRNA was confirmed. With the depletion of poly (A) tail, the protective effect of BBR on RB1 mRNA from degradation in the process of ischemia/reperfusion was also abolished, supporting the conclusion that the antagonist effect of BBR on Rb down-regulation in ischemia/reperfusion was due to its direct binding to the poly (A) tail of RB1 mRNA, which effectively stabilized the mRNA in this pathogenic process. Since BBR had a negative effect on the RB1 promoter and RB1 has no AU-rich elements (ARE) in 3′UTR sequences [48], it could be concluded that the poly (A) tail is one of the primary target of BBR regulating the level of Rb protein in the processes of ischemia/reperfusion.

Previous *in vitro* experiment has reported that BBR prefer to poly (A) isolated from *Escherichia coli* strain than the tRNA [49]. Another study has shown that BBR could induce self-structure formation with in poly(A) sequences *in vitro*, predicting that could be used to develop new types of RNA-based therapeutic interventions for viral infections [50]. However, our present experiments have shown that BBR could act on the poly (A) tail located in the 3′UTR and inhibit the degradation of RB1 mRNA subsequently stabilizing Rb translation, suggesting that the BBR-poly (A) effect takes place differently under *in vitro* conditions and in living cells.

The regulation of BBR in cell cycle has been studied in tumor cells. BBR can dose and time dependently arrest tumor cells at G0/G1 phase, or in G2/M phase though its function on p53, cyclins, CDKs pathways, suppressing the proliferation of tumor cell. Cells with low concentrations (12.5–50 µM, 4.69–18.75 µg/ml) of BBR is more in GO/G1 phase and high concentrations (>50 µM, 18.75 µg/ml) of BBR is more in G2 phase [51]–[54]. BBR could also induce cancer cell apoptosis by upregulating apoptotic factors [55]–[57]. However, the dosage of BBR used in cancer cell studies ranges from 10 µM to 75 µM (3.75 µg/ml–28.12 µg/ml), which is about seven times to five hundred times as that dosage used in the present study, believing they worked as a toxic dosage to living cells [58], [59]. The dosage of BBR used in this study was 1.34 µM (equal to 0.5 µg/ml). In such low concentration, BBR limited the impact on normal cells in toxicity. However, in ischemic injury, BBR showed the activity clearly. Particularly, BBR worked on the regulation of Rb, which enabled the cells to arrest at G0/G1 phase, while protected the neurons from the damage. This would be of interesting that whether the role of Rb in cell cycle regulation and the effect of BBR in the ischemic damage are also applicable to other tumor cell lines.

In conclusion, the protective effect of BBR on neural damage was found to be dependent on the inhibition of degradation of RB1 mRNA. BBR could bind to the poly(A) tail on RB1 mRNA to antagonize the degradation of RB1 mRNA and the up-regulation of Rb protein during ischemia/reperfusion, which in tune controls the transcription factors releasing, arrest the cell cycle, inhibit apoptosis, and facilitate cell survival in the injury process. For the popularity of poly(A) tail in the transcriptome, the poly(A) tail-dependent protective effect of BBR on neurons suffering ischemia/reperfusion sheds new light on the understanding of BBR pharmacological activities.
